# Swapping the N- and C-terminal domains of human apolipoprotein E3 and AI reveals insights into their structure/activity relationship

**DOI:** 10.1371/journal.pone.0178346

**Published:** 2017-06-23

**Authors:** Mark T. Lek, Siobanth Cruz, Nnejiuwa U. Ibe, Wendy H. J. Beck, John K. Bielicki, Paul M. M. Weers, Vasanthy Narayanaswami

**Affiliations:** 1 Department of Chemistry and Biochemistry, California State University Long Beach, Long Beach, California, United States of America; 2 Donner Laboratory, Lawrence Berkeley National Laboratory, Berkeley, California, United States of America; Beijing Key Laboratory of Diabetes Prevention and Research, CHINA

## Abstract

Apolipoprotein (apo) E3 and apoAI are exchangeable apolipoproteins that play a dominant role in regulating plasma lipoprotein metabolism. ApoE3 (299 residues) is composed of an N-terminal (NT) domain bearing a 4-helix bundle and a C-terminal (CT) domain bearing a series of amphipathic α-helices. ApoAI (243 residues) also comprises a highly helical NT domain and a less structured CT tail. The objective of this study was to understand their structural and functional role by generating domain swapped chimeras: apoE3-NT/apoAI-CT and apoAI-NT/apoE-CT. The bacterially overexpressed chimeras were purified by affinity chromatography and their identity confirmed by immunoblotting and mass spectrometry. Their α-helical content was comparable to that of the parent proteins. ApoE3-NT/apoAI-CT retained the denaturation profile of apoE3 NT domain, with apoAI CT tail eliciting a relatively unstructured state; its lipid binding ability improved dramatically compared to apoE3 indicative of a significant role of apoAI CT tail in lipid binding interaction. The LDL receptor interaction and ability to promote ABCA1-mediated cholesterol efflux of apoE3-NT/apoAI-CT was comparable to that of apoE3. In contrast, apoAI-NT/apoE-CT elicited an unfolding pattern and lipid binding ability that were similar to that of apoAI. As expected, DMPC/apoAI-NT/apoE-CT discoidal particles did not elicit LDLr binding ability, and promoted SR-B1 mediated cellular uptake of lipids to a limited extent. However, apoAI-NT/apoE-CT displayed an enhanced ability to promote cholesterol efflux compared to apoAI, indicative of a significant role for apoE CT domain in mediating this function. Together, these results indicate that the functional attributes of apoAI and apoE3 can be conferred on each other and that NT-CT domain interactions significantly modulate their structure and function.

## Introduction

Apolipoprotein E3 (apoE3) and apolipoprotein AI (apoAI) are exchangeable apolipoproteins that are key players in cardiovascular disease predominantly due to their abilities to maintain and regulate plasma triglyceride and cholesterol homeostasis [1–4]. Both show broadly similar functional characteristics with an ability to bind and transport lipids as large lipoprotein complexes, which serve as a vehicle for other amphipathic and hydrophobic proteins, lipid and nucleic acid moieties in an aqueous environment such as the plasma. Nonetheless, there are critical differences between the two from a functional, molecular and physiological perspective.

ApoE is considered anti-atherogenic predominantly due to its ability to interact with lipoproteins and serve as a ligand for the low density lipoprotein receptor (LDLr) family of proteins, a process that leads to receptor mediated endocytosis and consequent lowering of atherogenic lipoproteins and their clearance from plasma. There is a body of evidence that established this view, with early studies demonstrating that apoE-null mice exhibit massive accumulation of remnant lipoproteins and develop severe atherosclerosis [5]; conversely, transgenic mice over expressing apoE manifest marked resistance to diet-induced hypercholesterolemia [6]. In addition, apoE’s role gains prominence during atherosclerosis, when it participates in reverse cholesterol transport with macrophages secreting large amounts of lipid-free apoE [7], which in turn promotes cholesterol efflux and formation of high density lipoproteins (HDL) containing apoE. In the plasma, apoE resides predominantly on VLDL, chylomicron remnants and a sub species of HDL; in the brain, it is one of the major apolipoproteins that has been identified to play a crucial role in brain cholesterol metabolism, and is located on HDL-like particles [8].

ApoAI is considered atheroprotective primarily due to its role in promoting cholesterol efflux from macrophages and in modulating immune cell response [9]. In humans, apoAI deficiency is associated with coronary heart disease [10], and like apoE, transgenic mice overexpressing apoAI are protected against atherogenesis [11]. It is the main protein component of plasma HDL, the levels and functionality of which are associated with protection against atherosclerosis. ApoAI plays a critical role in HDL metabolism participating in the process of particle maturation such as activation of lecithin cholesterol acyltransferase leading to formation of cholesterylester core and in eventual delivery of cholesterylester via interaction with SR-BI located on sites such as liver and adrenal tissue. As a high-affinity lipid binding protein, its ability to promote ATP-binding cassette A1 (ABCA1)-mediated cholesterol and phospholipid efflux is associated with the lipid-free or lipid-poor form of apoAI, which results in biogenesis of the nascent HDL particle.

In humans the apoE gene is polymorphic with three major alleles: *APOE* ε2, *APOE* ε3, and *APOE* ε4 coding for apoE2, apoE3, and apoE4 isoforms, respectively. These isoforms differ only at the amino acid at positions 112 and 158 in the NT domain: apoE3 contains a Cys and Arg, respectively at these locations, while apoE2 contains Cys, and apoE4 has Arg at both locations. While apoE3 is considered anti-atherogenic, apoE4 has been associated with cardiovascular disease and is a major risk factor for Alzheimer’s disease [2, 12]. ApoE3 is a 34 kDa protein (299 residues) that is organized as a 22 kDa N-terminal (NT) domain (residues 1–191) with four amphipathic α-helices organized in an anti-parallel manner as a helix bundle, and, a 10 kDa C-terminal (CT) domain (residues 201–299) that also bears a series of amphipathic α-helices; the 2 domains are linked by a flexible protease sensitive hinge. X-ray crystal analysis of apoE3(1–191) reveal that the α-helices of the NT domain are arranged such that the hydrophobic residues of each helix are localized towards the interior, and the polar residues face the exterior aqueous environment [13]. The helix bundle is stabilized by hydrophobic interactions (including aromatic stacking and leucine zippers) and intra- and inter-helix salt bridges. Together, they impose tremendous stability on this domain with the free energy of unfolding of 10–12 kcal/mol [14] and a mid-point of denaturation of ~ 2.5 M guanidine-HCl (GdnHCl) [15]. When associated with a lipid surface, helix 4 of the NT domain, which has an abundance of positively charged residues, serves as the high-affinity ligand for binding the LDLr along with Arg 172 [16]. The NT domain retains its stability, lipid binding capability and ability to interact with the LDLr in isolation. The helices of the CT domain have a high affinity for lipid binding and promote apoE3 oligomerization [17]. Within its CT domain, apoE3 also bears a potent ability to promote ABCA1-mediated cholesterol efflux, a feature that is under intense scrutiny for development of cholesterol-lowering peptide mimetic agents [18–22]. NMR analysis of a variant form of apoE3 bearing multiple mutations at the CT domain (F257A/W264R/V269A/L279Q/V287E) [23] also reveal a 4-helix bundle in the NT domain; in addition, it shows that the CT helices C1, C2 and C3 are wrapped around the helix bundle. While it is possible that the apposition of the CT domain with the NT domain is a consequence of the engineered mutations, it is not known if the interaction between the two domains alters the functional behavior of the individual domains. To obtain further insight into this interaction, a chimera was designed in the current study by swapping the CT tail with that from the structurally closely related apoAI.

Having evolved from a common ancestral gene, both apoE and apoAI show similar structural characteristics, characterized by Pro-punctuated tandem repeats of 22-mers [24] that form amphipathic α-helices [25, 26]. Although a high-resolution structure of apoAI is not available, there are several lines of evidence from a variety of biophysical studies [9] which suggests that the NT domain bears a 4-helix bundle, similar to apoE3. However, the NT domain of apoAI is not as stable as that of apoE3, with a free energy of unfolding of 2–4 kcal/mol, and a mid point of denaturation of ~ 1.0 M GdnHCl [14]. Further, truncation of residues 185–243 results in an NT domain that appears to be a double belt [27], as noted by X-ray analysis. The notion that the structure of the isolated NT domain may not resemble that in the context of the intact protein, raises questions about the dependency of this domain on the CT end of the protein from a functional perspective. Helices 1, 9 and 10 are believed to play a major role in high-affinity lipid binding of apoAI, while helix 10 has been reported to be involved in ABCA1-mediated cholesterol efflux capability of the protein. To gain further understanding of the interaction between these two domains, a second chimera was designed by swapping the CT tail of apoAI with that from apoE3. The objective of the current study was to examine the structure-function behavior of apoE3 and apoAI, each NT domain bearing the CT tail of the other.

## Materials and methods

### Design and generation of chimeric DNA constructs

**Fig 1:**
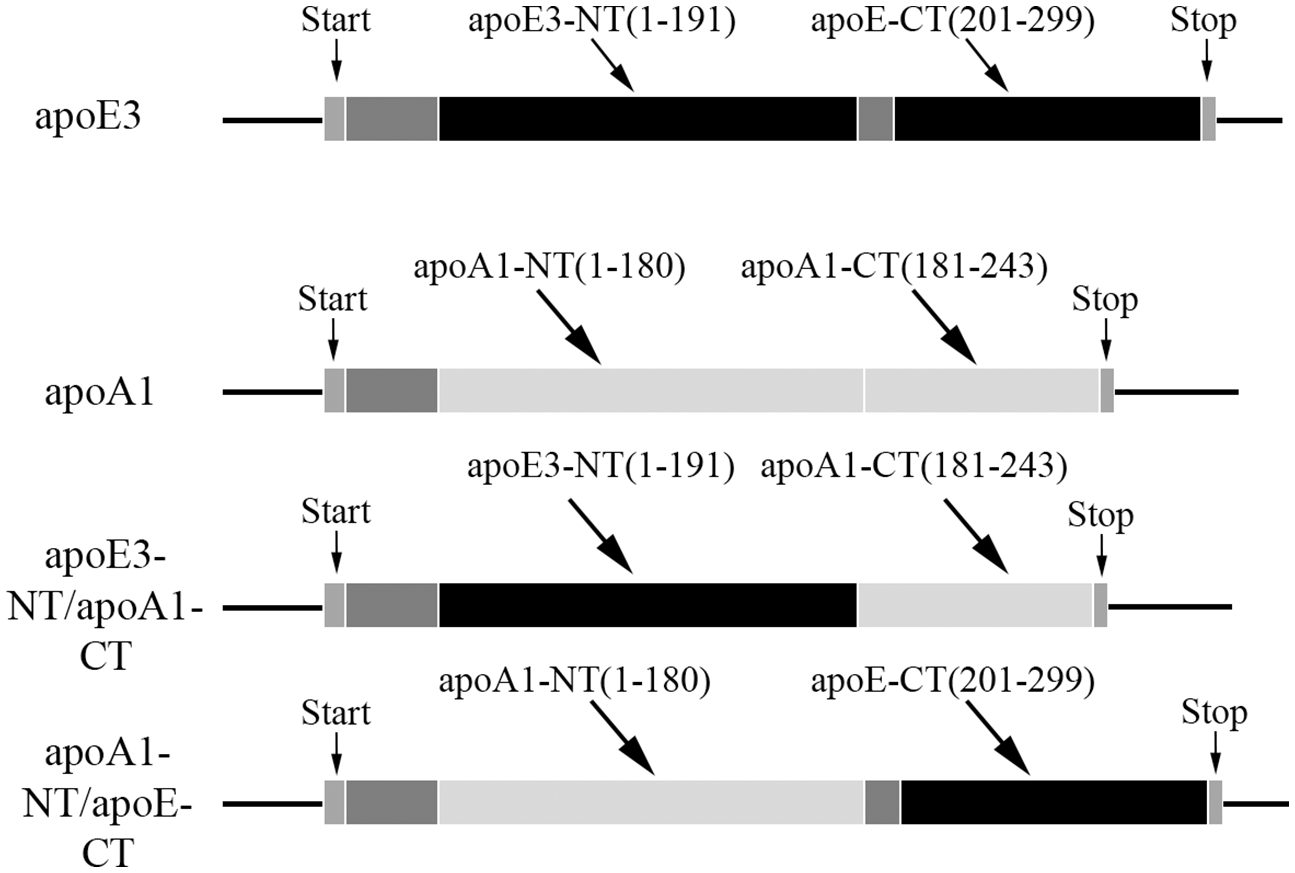
Schematic representation illustrating design and generation of apoE3-NT/apoAI-CT and apoAI-CT/apoE-NT chimeras. The NT and CT domains of the parent apoE3 (black) and apoAI (light grey) are shown; the boundaries for the individual domains represent consensus from various labs: apoE3 NT domain (residues 1–191) and apoE CT domain (201–299), with residues 192–200 encompassing the protease sensitive linker loop; apoAI NT domain (residues 1–185) and CT domain (residues 186–243). The apoE3-NT/apoAI-CT chimera encompassed residues 1–191 from apoE3 and 181–243 from apoAI, while apoAI-NT/apoE-CT chimera was composed of residues 1–180 from apoAI and 192–299 from apoE. The start and stop sites of the coding region are indicated. A His-tag and protease cleavage sites (dark grey) were placed immediately upstream of the first residue. For sake of convenience and clarity, residue numbering of the parent proteins was retained for the chimeras.

Two chimeric apolipoproteins were generated (Fig 1): one bearing the NT domain of apoE3 and the CT domain of apoAI, referred to as apoE3-NT/apoAI-CT, and the other bearing the NT domain of apoAI and the small linker loop and the CT domain of apoE3, referred to as apoAI-NT/apoE-CT (the CT domain of apoE is shown without the isoform designation since the amino acid sequence is identical for the apoE isoforms in this domain). The two parent wild type and two chimeric constructs were housed in a pET-20b(+) vector containing ampicillin and chloramphenicol resistance genes for increased target expression and bear a hexa-His tag at the N-terminal end to facilitate purification. The codon-optimized sequence of apoE3-NT/apoAI-CT was generated by the overlap extension polymerase chain reaction method using overhang primers [28, 29] (details of the amplification step and ligation are provided in (**Fig A and Table I in** S1 File).

### Overexpression and purification of chimeric and parent apolipoproteins

The pET-20b(+) plasmid bearing the coding sequence for apoE3, apoAI, apoE3-NT/apoAI-CT or apoAI-NT/apoE-CT was used to transform *E*. *coli* BL21-Gold (DE3) pLys competent cells (Agilent Technologies, Santa Clara, CA). The proteins were overexpressed, isolated and purified as described previously [30]. In select cases (apoAI and apoAI-NT/apoE-CT), the affinity–purified proteins were subjected to gel filtration chromatography using Superdex 200 (Sigma-Aldrich, St. Louis, MO) packed in an XK-26/70 column (GE Healthcare Life Sciences, Piscataway, NJ) at a flow rate of 0.7 ml/min.

The purified proteins were quantified by UV-Vis spectrometry using a NanoDrop 2000 UV-Vis spectrophotometer (Thermo Scientific, Wilmington, DE) using the following molar extinction coefficient at 280 nm: apoE3-NT/apoAI-CT: 30,940; apoAI-NT/apoE-CT: 47,440; apoE3: 45,950; and, apoAI: 33,920 M^-1^cm^-1^ or by the D_c_ protein assay kit (Bio-Rad Laboratories Company, Hercules, CA). The purity of the samples was assessed by SDS-PAGE using 4–20% acrylamide gradient Tris-glycine gels (Invitrogen Life Technologies, Carlsbad, CA) under reducing or non-reducing conditions. For Western blot analysis, HRP conjugated polyclonal goat anti-human apoAI or anti-human apoE antibody (Abcam Company, Cambridge, MA) was employed at a 1:3000 or 1:4000 dilution, respectively. The bands were visualized using Amersham ECL Plus Western Blotting Detection Reagent (GE Healthcare Life Sciences, Piscataway, NJ).

### Secondary structural analysis

The secondary structure of the chimeric proteins was assessed by far UV circular dichroism (CD) spectroscopy. The details of the CD experiments are described in S1 File.

### Guanidine-HCl-induced unfolding

GdnHCl induced denaturation studies were performed to determine protein stability and unfolding pattern, and the percent maximal change was calculated from the ellipticity value at 222 nm as described previously [31]. About 0.2 mg/ml protein was incubated for 16 h at 24°C in the presence of increasing concentration (0 to 6 M) of ultra-pure grade GdnHCl (MP Biomedicals, Solon, OH) and 5x molar excess of tris(2-carboxyethyl)phosphine (TCEP) in 10 mM ammonium bicarbonate, pH 7.4. The midpoint of denaturation (the concentration of GdnHCl required to cause a 50% decrease in the maximal change) [GdnHCl]_1/2_ was determined for each construct.

### Fluorescence spectroscopy

The binding of 1-anilinonaphthalene-8-sulfonate (ANS) to the chimeric and parent proteins was monitored by following the fluorescence emission spectra of 250 μM ANS in 10 mM ammonium bicarbonate, pH 7.4 in the absence or presence of 5 μM protein at 24°C. Fluorescence measurements were made in a Perkin-Elmer spectrofluorometer (model LS 50B). An average of 3 scans were recorded for 3 freshly folded samples, at a scan speed of 100 nm/min. The excitation wavelength was set at 395 nm, and the emission spectra were scanned between 400 and 600 nm (excitation and emission slit widths at 6 nm).

### Lipid binding assay

The ability of the chimeric apolipoproteins to bind lipids and cause vesicle solubilization was determined as described previously [32] using multilamellar vesicles (MLVs) of 1,2-Dimyristoyl-*sn*-glycero-3-phosphocholine (DMPC) (Avanti Polar Lipids, Alabaster, AL), with slight modifications (details provided in S1 File). The time required for initial absorbance to decrease by 50% (T_1/2_) and the rate constant (k, reciprocal of T_1/2_) were determined for the chimeras and the parent proteins.

Preparation of DMPC- and DMPC/DiI-bound complexes of chimera or parent proteins is described in S1 File.

### LDLr binding assay

The ability of the lipid-associated chimeras to bind to the soluble LDLr with a c-Myc epitope was assessed using a co-immunoprecipitation assay as described previously [32, 33] with 0 or 10 μg DMPC/chimera complexes, 10 μg of sLDLr, 2 mM Ca^+2^ in PBS. ApoE was detected by Western blot using HRP-conjugated polyclonal apoE antibody. A replica experiment was conducted wherein an anti-c-Myc antibody (9E10) was utilized.

### Cellular uptake of DMPC/chimera particles by glioblastoma cells

Cellular uptake of DMPC-bound complexes of chimera was determined using human glioblastoma cell line A-172 [34] (details provided in S1 File). Human brain A-172 glioblastoma cells were obtained from ATCC (Manassas, VA).

### Cholesterol efflux assay

The ability of apolipoproteins to mediate cellular cholesterol efflux was assessed using J774 mouse macrophages. Cells were treated with and without a cAMP analog to modulate ABCA1 expression as described previously [19]; apolipoprotein acceptors were used in lipid-free form (details provided in S1 File).

### Statistical analysis

Where appropriate, data were expressed as mean ± SD of at least three independent experiments and statistical analyses performed using Student’s t-test.

## Results

### Design and characterization of chimeric apolipoproteins

To determine the structural basis of the functional differences between the NT and CT domains of apoE3 and apoAI, chimeric apolipoproteins were generated by swapping the CT domain of each other, Fig 1. The splice sites broadly aligned with the limits of the domain organization of each apolipoprotein. The DNA construct of apoE3-NT/apoAI-CT codes for residues 1–191 of apoE3 immediately followed by residues 181–243 of apoAI (**Fig A** and details in S1 File), while that of apoAI-NT/apoE-CT codes for residues 1–180 of apoAI followed by 192–299 of apoE3. In both cases the nucleotide sequence was verified to confirm the presence of the desired segments of the parent proteins in the spliced DNA.

**Fig 2:**
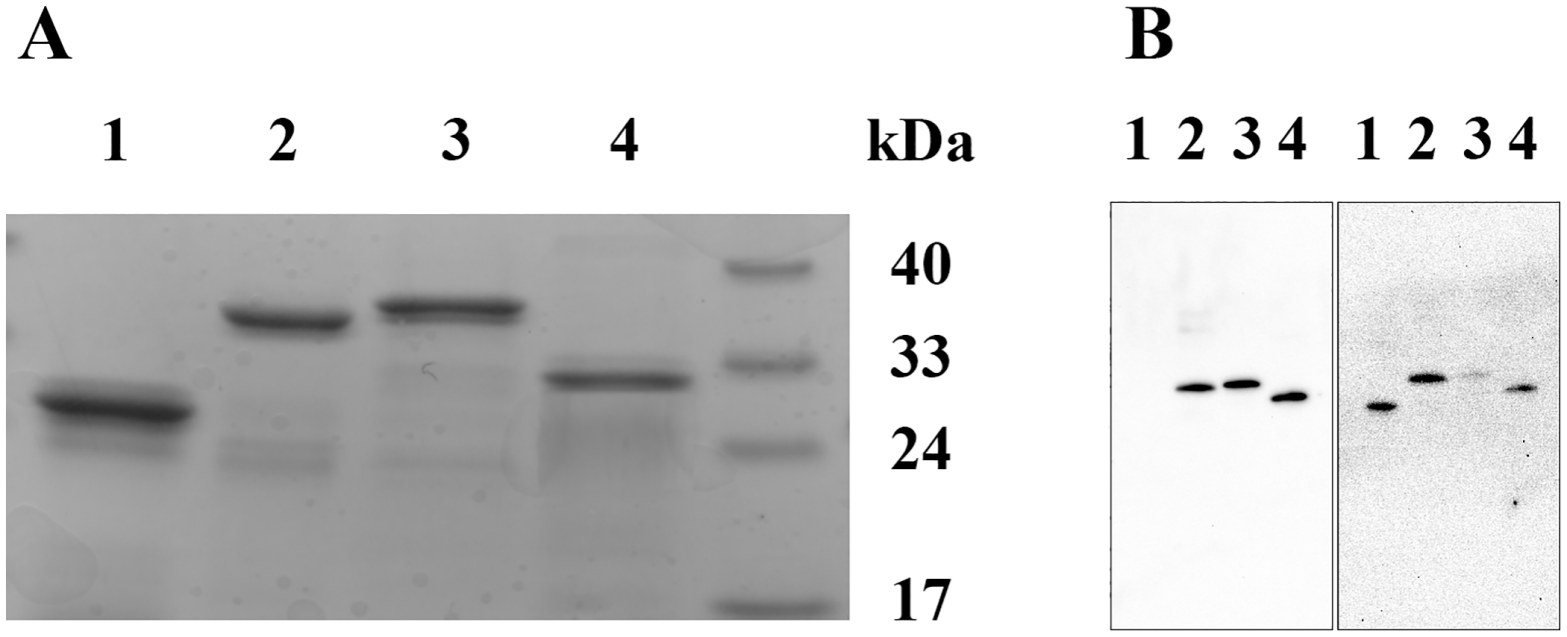
Characterization of chimeric apolipoproteins. **Panel A**. SDS-PAGE analysis of the chimeric apolipoproteins. Electrophoresis of chimeric and parent proteins (20 μg protein) was carried out using a 4–20% acrylamide gradient Tris-glycine gel under reducing conditions in the presence of BME. **Panel B**. Western blot analysis of chimeras (0.5 μg protein) using mouse HRP-conjugated apoE polyclonal antibody (*Left*) or apoAI antibody (*Right*). Lane assignments are as follows: Lane 1, apoAI; Lanes 2, apoAI-NT/apoE-CT; Lanes 3, apoE3; Lanes 4, apoE3-NT/apoAI-CT.

SDS-PAGE analysis of the chimeric and their parent proteins under reducing conditions, Fig 2**, Panel A**, revealed molecular masses of ~32 and 35 kDa for apoE3-NT/apoAI-CT and apoAI-NT/apoE-CT, respectively, (expected masses: 30,975 and 34,864 Da, respectively). This suggests that the chimeras contain the designed individual components of the parent apolipoproteins. The molecular masses of apoE3 and apoAI were ~ 34 and 29 kDa, respectively. Western blot analysis using mouse HRP-conjugated apoE polyclonal antibody, Fig 2, **Panel B**, *Left*, yielded robust bands at ~32 and 35 kDa for apoE3-NT/apoAI-CT and apoAI-NT/apoE-CT, respectively, confirming the presence of apoE3 epitopes in both chimeras. The absence of a band for apoAI confirms that the apoE polyclonal antibody does cross react with apoAI.

When mouse HRP-conjugated apoAI polyclonal antibody was used, Fig 2, **Panel B,** *Right*, a strong band of ~ 35 kDa was noted for apoAI-NT/apoE-CT, confirming the presence of apoAI epitope in this chimera. A band (albeit less robust) was noted for apoE3-NT/apoAI-CT, which confirms the presence of the apoAI epitope in this chimera as well. Taken together, the nucleotide sequence, SDS-PAGE and Western blot confirm the presence and correct splicing of the desired swapped domain components in the two chimeras, and the purity of the preparations.

### Secondary and tertiary structural analysis, GdnHCl-induced unfolding and cross-linking studies

CD spectroscopy was carried out to assess the secondary structure of the chimeras. The ellipticity of both chimeras was measured in the far UV range and compared with those of the parent proteins (**Fig B in** S1 File**);** both reveal profiles with troughs at 208 and 222 nm, a signature feature of an α-helix, similar to that noted for the parent proteins. The α-helical content for both apoE3-NT/apoAI-CT and apoAI-NT/apoE-CT was 45%, while that for apoE3 and apoAI was ~ 40%, the differences noted were within the error limit of CD measurements and variations between preparations.

**Fig 3:**
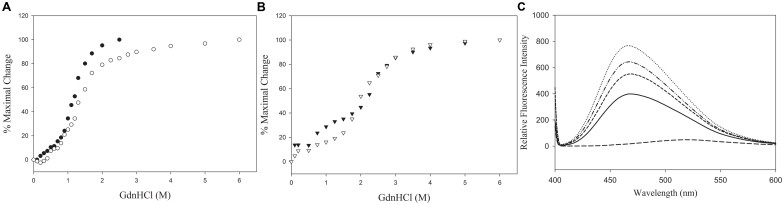
Unfolding and folding behavior of chimeras. **Panels A and B**. GdnHCl-induced denaturation profiles of apoAI-NT/apoE-CT (**A**) and apoE3-NT/apoAI-CT (**B**). The samples (0.2 mg/ml) were incubated with increasing concentration of GdnHCl and 5x molar excess of TCEP for 16 h at 24°C. The ellipticity value at 222 nm was measured and protein unfolding plotted as % maximal change in which 100% represents completely unfolded protein. ApoAI (filled circles); apoAI-NT/apoE-CT (open circles); apoE3 (closed inverted triangles); and apoE3-NT/apoAI-CT (open inverted triangles). **Panel C**. ANS fluorescence emission spectra of chimeric and parent proteins. About 50 μg of each protein sample was excited at 395 nm and the emission spectra were recorded at 100 nm/min from 400 to 600 nm. ApoAI (_____); apoAI-NT/apoE-CT (·······); apoE3 (-------); apoE3-NT/apoAI-CT (··-··-··) and ANS (__ __ __).

GdnHCl-induced denaturation was carried out to examine the effect of appending the CT tail of one on the NT domain of the other apolipoprotein and to obtain an estimate of the overall protein stability, in comparison with that of the parent proteins under reducing conditions, Fig 3**, Panels A and B**. ApoAI shows a single transition with a midpoint of denaturation (concentration of GdnHCl required to cause a 50% decrease in the maximal change, [GdnHCl]_1/2_) of ~1.2 M. The unfolding behavior of the chimeric apoAI-NT/apoE-CT ([GdnHCl]_1/2_ of ~1.3 M) was broadly similar to that of apoAI. In contrast apoE3 shows a biphasic denaturation profile [35, 36], with the first transition attributed to the unfolding of the CT domain ([GdnHCl]_1/2_ of ~0.75 M) followed by that of the NT domain ([GdnHCl]_1/2_ of ~2.50 M). The denaturation profile of apoE3-NT/apoAI-CT showed a biphasic pattern as well; however, the initial transition showed a lower [GdnHCl]_1/2_ of ~ 0.1 M, reflective of a segment highly susceptible to denaturation, likely corresponding to the unfolding of apoAI CT tail; the second transition showed a strong overlap corresponding to the unfolding of apoE3 NT domain ([GdnHCl]_1/2_ of ~ 2.6 M GdnHCl). This suggests that the individual domains of the apoE3-NT/apoAI-CT chimera recapitulate their fold in the context of their parent proteins. Under non-reducing conditions, the unfolding behavior of both chimeras did not alter significantly from that noted under reducing conditions (data not shown).

To determine if swapping the CT domains altered the overall fold or exposure of hydrophobic core segments of the protein, ANS fluorescence emission was monitored, Fig 3**, Panel C**. ANS is an amphiphilic probe that fluoresces upon binding to hydrophobic pockets or cores in proteins leading to changes in the wavelength of maximal fluorescence emission (λ_max_) and to an enhancement in emission intensity [37]. In buffer, ANS was relatively non-fluorescent with a λ_max_ of 519 nm; in the presence of apoE3, a large increase in ANS fluorescence emission was noted, with a λ_max_ of ~467 nm, indicative of a folded state; swapping its CT domain with that from apoAI resulted in a small increase in intensity by ~18%, with the λ_max_ remaining at ~467 nm. In the case of apoAI as well, a large increase in intensity compared to ANS alone was noted, which was accompanied by a blue shift to 468 nm; upon swapping the CT domain of apoAI with that from apoE, the λ_max_ remains at 466 nm while the intensity increased further by almost 2-fold. The increase was likely due to the presence of the structured segment of apoE CT tail (107 residues), although we cannot exclude the possibility of the contribution of the larger surface area it offers compared to apoAI CT tail (62 residues).

### Lipid binding characteristics

**Fig 4:**
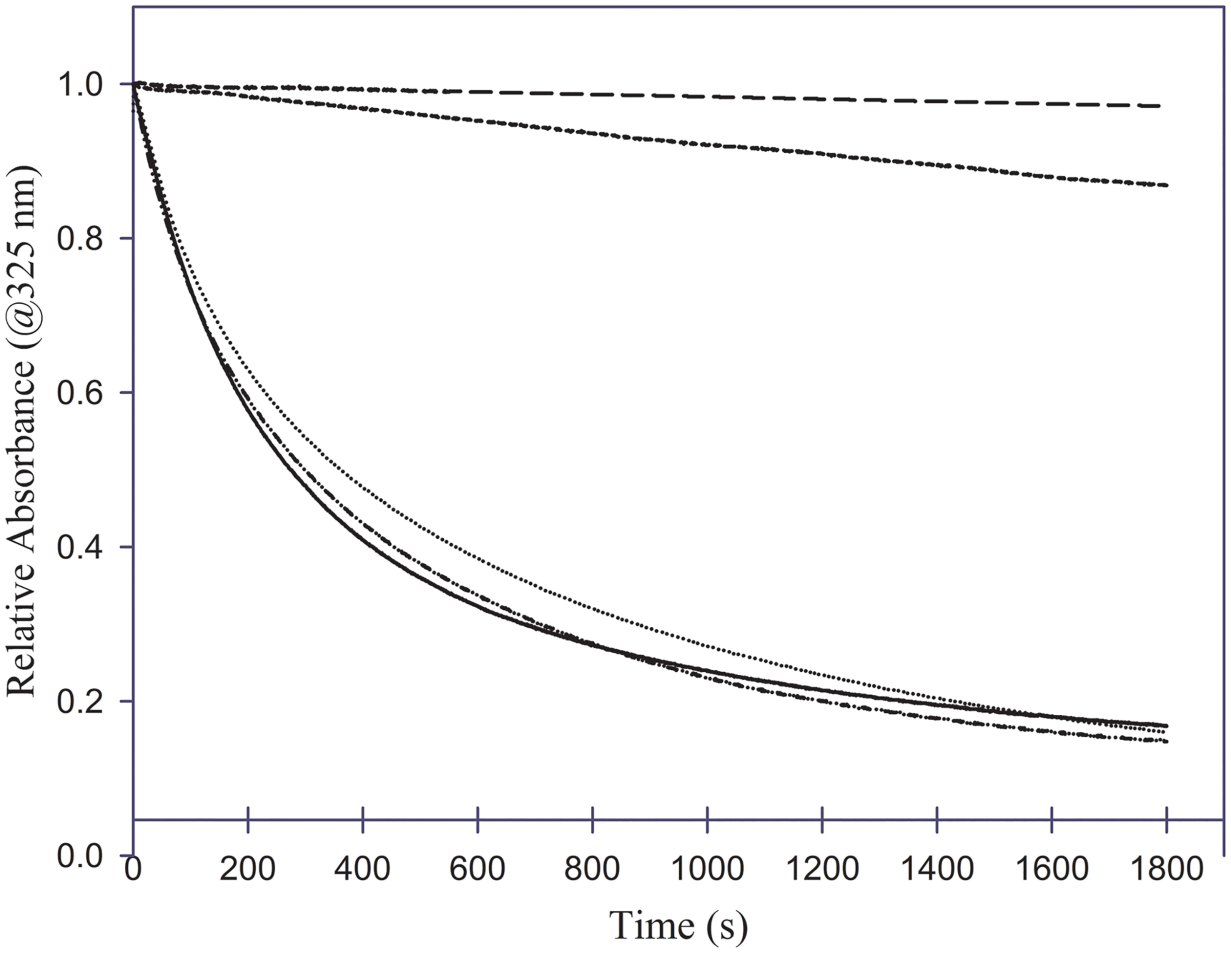
Phospholipid vesicle solubilization capability of chimeras. About 125 μg of DMPC MLVs were equilibrated in 400 μl of PBS in a cuvette at 23.7°C in a Peltier-controlled spectrophotometer. Vesicle solubilization was initiated by addition of 125 μg of apolipoprotein, mixed rapidly and the change in absorbance at 325 nm measured for 30 min. Data were normalized to initial absorbance immediately following addition of protein. ApoAI (_____); apoAI-NT/apoE-CT (·······); apoE3 (-------); apoE3-NT/apoAI-CT (··-··-··); and DMPC vesicles alone in the absence of apolipoproteins (__ __ __).

The lipid binding ability of the chimeras were compared to that of the parent proteins using the DMPC vesicle solubilization assay [32]. This assay involves incubation of apolipoproteins with MLVs prepared with DMPC at 1:1 ratio (w/w). At the transition temperature of DMPC (23.9°C), apolipoproteins typically convert the vesicular structures (~200 nm diameter) to small discoidal complexes (10–20 nm diameter), which is observed as a decrease in absorbance due to light scattering at 325 nm. Fig 4 shows a plot of change in absorbance at 325 nm as a function of time. ApoAI elicits a rapid decrease in absorbance with a T_1/2_ of ~ 260 s and *k* value 3.6 x 10^−3^ s^-1^ in agreement with previous studies carried out under similar conditions [38]. In contrast, apoE3 shows a very poor ability to solubilize the vesicles, with T_1/2_ of >1800 s. Such behavior by apoE3 and apoAI is has been noted by us and other researchers previously [32, 38, 39]. Interestingly, the T_1/2_ and *k* value for apoE3-NT/apoAI-CT chimera were 298 ± 3 s and 3.3 x 10^−3^ ± 3.2 x 10^−5^ s^-1^, respectively, which is comparable to that apoAI; and those for apoAI-NT/apoE-CT are ~ 319 ± 90 s and 3.3 x 10^−3^ ± 1.1 x 10^−3^ s^-1^, respectively.

It should be noted that prolonged overnight incubation of apoE3 with DMPC vesicles under these conditions eventually causes transformation of vesicular to discoidal structures, with the assumption that the entire apoE3 molecule interacts with phospholipids. These DMPC-bound complexes are discoidal in shape and are referred to as reconstituted HDL; they recapitulate all the functional features of native apoE3 in terms of their ability to bind and interact with the LDLr and facilitate cellular uptake of lipoprotein particles. The lipid/protein ratios, approximate molecular mass of the DMPC complexes, and estimated number of proteins per particle are shown in Table 1. In general, non-denaturing PAGE analysis shows that apoE3 forms larger reconstituted complexes compared to apoAI, **Fig C** in S1 File; complexes bearing the NT domain of apoE3 resulted in two bands (200,000–300,00 Da band with 3–4 proteins/particle, and ~500,000 Da band with 6–8 proteins/particle. In contrast, those with the NT domain of apoAI resulted in smaller bands of ~ 200,000 Da mass with 2–3 proteins/particle.

**Table 1:** Characterization of DMPC/chimera particle size and protein/lipid composition.

DMPC complex	Lipid/protein ratio (m/m)	Approximate Molecular mass (Da)	# of proteins/particle
ApoAI	40:1	200,000	3
ApoAI-NT/apoE-CT	100:1	200,000	2
ApoE3	70:1	300,000	4
		500,000	6
ApoE3-NT/apoAI-CT	50:1	200,000	3
		500,000	8

### LDLr binding ability and cellular uptake

**Fig 5:**
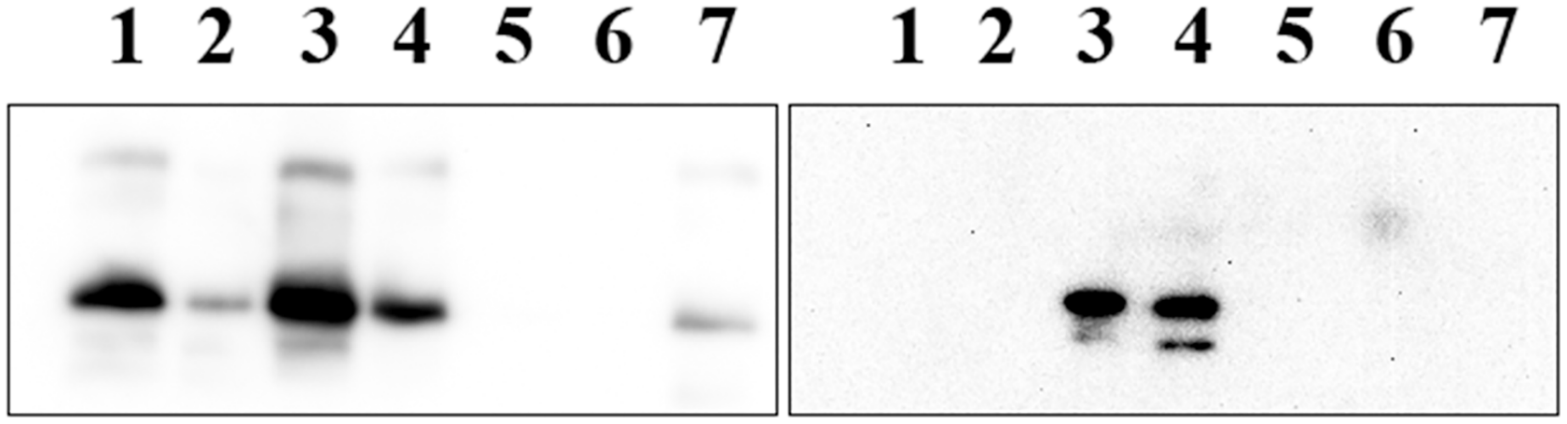
LDLr binding ability of chimeras. DMPC/chimera complexes (10 μg protein) were incubated with 10 μg of sLDLr, followed by co-IP with anti-c-Myc-agarose. sLDLr-bound apoE was detected by Western blot using HRP-conjugated polyclonal apoE antibody (*Top*); the lane assignments are: Lane 1, apoAI; lane 2, apoAI-NT/apoE-CT; lane 3, apoE3; lane 4, apoE3-NT/apoAI-CT; lane 5, apoAI added in the absence of sLDLr; lane 6, apoE3 added in the absence of LDLr; lane 7, LDLr alone in the absence of added proteins. A replica experiment was conducted wherein an anti-c-Myc antibody (9E10) was utilized to identify the presence of LDLr in each reaction (*Bottom*).

Blacklow and colleagues devised a convenient method to assess the LDLr binding capability of lipoproteins using a soluble extracellular segment of the receptor [33]. The soluble LDLr (sLDLr) used in our study is composed of consecutive LA modules LA3-LA6 bearing a c*-*Myc tag that displays all the functional features of receptor-ligand binding. We have used this construct previously to capture the LDLr/rHDL complex [40, 41] and detect apoE via Western blot analysis. We deployed the same approach to initially assess the functionality of the lipid-associated forms of the chimeras in terms of their ability to recognize and bind the sLDLr. Western blot analysis using anti-c-Myc antibody to detect the presence of LDLr showed a band in all samples incubated with LDLr, Fig 5, confirming the presence of the receptor in all incubations. The absence of a band for apoE3 incubated without LDLr confirmed no cross reactivity between the anti-c-Myc-agarose antibody with apoE3. Of the two chimeras generated, only that which had the NT domain of apoE3 (apoE3-NT/apoAI-CT) displayed sLDLr-binding ability, as expected. This observation indicated that the presence of the CT domain of apoAI did not alter the ability of the apoE3 NT domain to bind lipid and attain the proper conformation to recognize and dock with the ligand binding module of the LDLr. It is known that even isolated apoE3 NT domain is capable of binding the LDLr in its lipid-bound state. Our findings further showed that the nature of the CT tail is irrelevant for the LDLr binding capability of apoE3. The apoAI-NT/apoE-CT chimera does not elicit any sLDLr binding ability.

**Fig 6:**
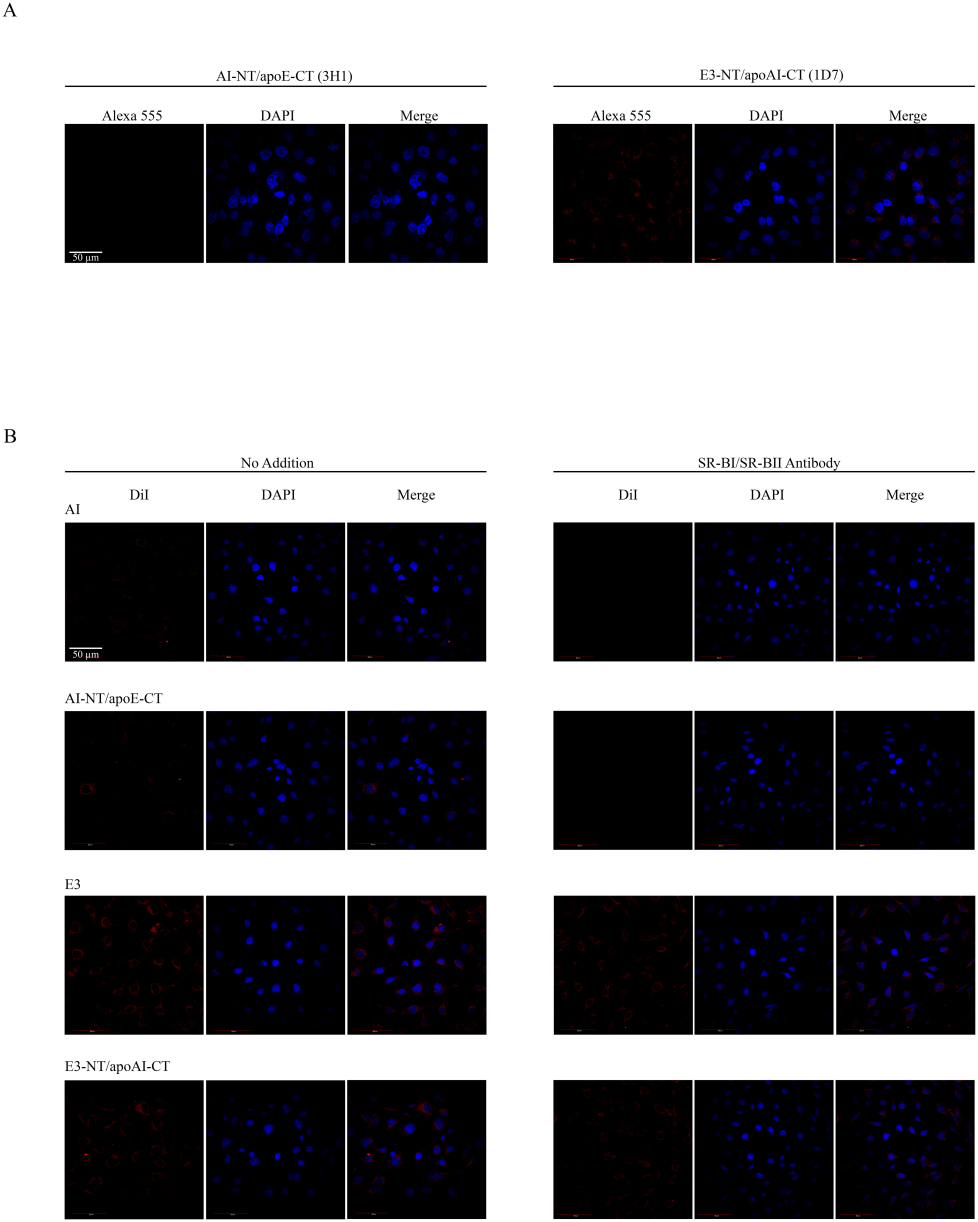
Uptake of rHDL/chimeras by glioblastoma cells. **Panel A. Uptake of apoE component of rHDL/chimera monitored by immunofluorescence.** DMPC/chimera complexes (10 μg/ml) were incubated with glioblastoma cells for 2h at 37°C. Cellular uptake of the lipoprotein particles was followed by immunofluorescence under a confocal laser scanning microscope using monoclonal antibodies against apoE CT domain (3H1) or NT domain (1D7) and Alexa555-labeled secondary antibody. **Panel B**. **Uptake of lipid component of rHDL/DiI/chimera monitored by direct fluorescence**. The chimeric and parent proteins were reconstituted with DMPC and DiI as described under S1 File. DMPC/DiI/chimera or parent complexes (0.5 μg/ml) were incubated with cells as above in the absence or presence of SR-B1/SR-BII antibody (1:500 dilution) and cellular uptake of the lipoprotein particles followed by direct fluorescence at 559 nm. The cells were stained with DAPI to visualize the nuclei. The scale bar represents 50 μm.

Subsequently, the ability of the chimeras to be internalized by A-172 glioblastoma cells was evaluated. Initial experiments were aligned with the sLDLr binding results above, wherein apoE-facilitated cellular uptake was followed by immunofluorescence using 1D7 and 3H1 monoclonal antibody, to detect the NT and CT domains of apoE, respectively, Fig 6A. DMPC/apoE3-NT/apoAI-CT chimera was internalized in a robust manner, showing punctate peri-nuclear endocytic vesicles. No visible uptake of DMPC/apoAI-NT/apoE-CT was observed, in agreement with the lack of the NT domain of apoE3 that houses the LDLr and proteoglycan binding sites. While this strongly suggested the involvement of the LDLr in the case of apoE3-NT containing particles, we cannot rule out the possibility of the involvement of HDL receptors such as SR-B1 in the binding and uptake of lipids. Therefore, in a complementary approach, we followed the uptake by labeling the lipid component of the lipoprotein complexes by using DMPC/DiI/chimeras or DMPC/DiI/parents in the absence and presence of SR-BI/SR-BII antibody, Fig 6B. DiI fluorescence was noted to a limited extent in cells treated with lipid-associated apoAI or apoAI-NT/apoE-CT; in the presence of SR-B1/SR-BII antibody, the intracellular DiI fluorescence was abolished. In contrast, cells treated with DMPC/DiI/apoE3 and DMPC/DiI/apoE3-NT/apoAI-CT displayed significant intra-cellular DiI fluorescence. In the presence of SR-BI/SR-BII antibody, the DiI fluorescence appeared to have decreased to a limited extent.

### Cholesterol efflux

**Fig 7:**
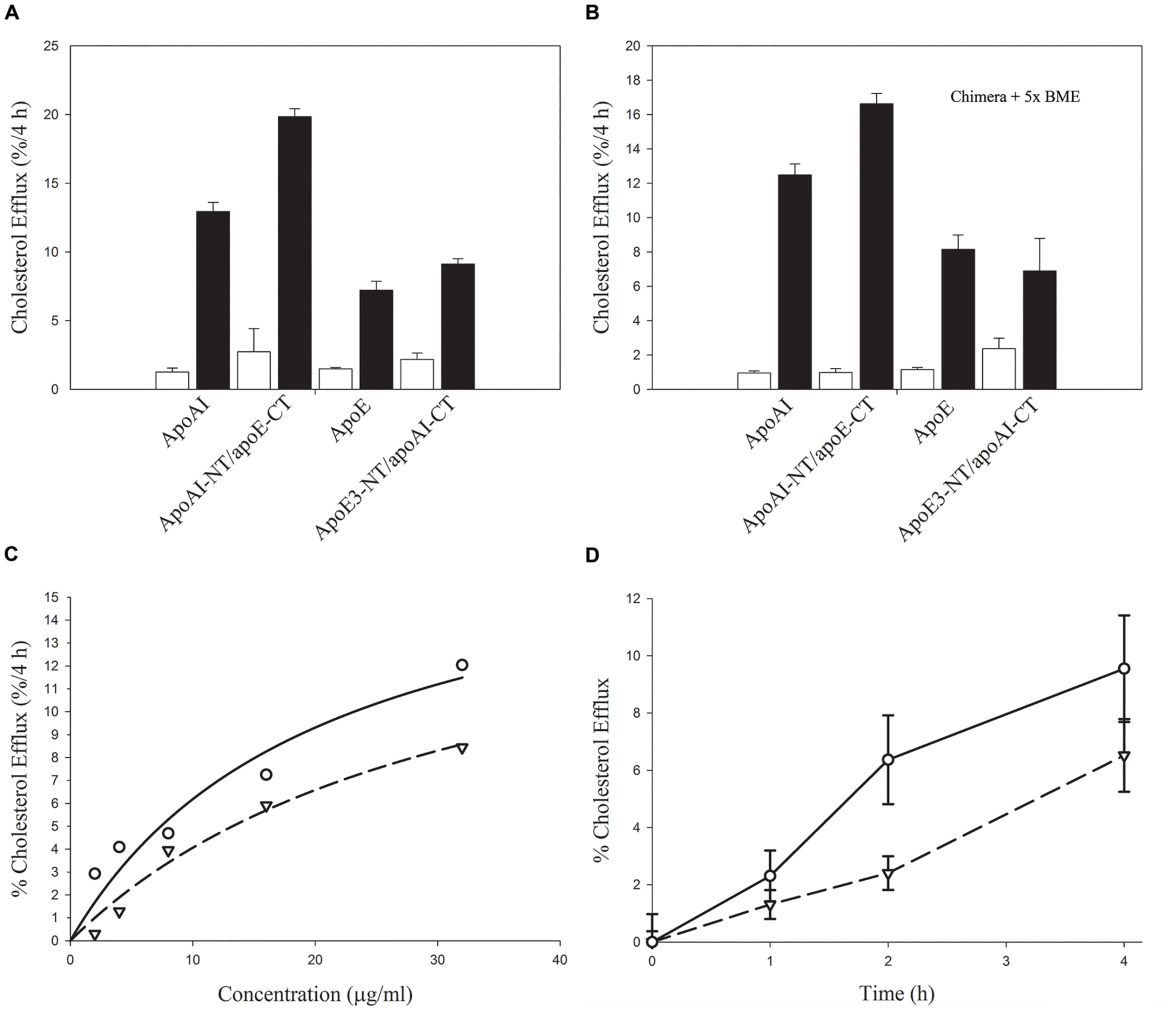
Chimeric apolipoproteins mediated cholesterol efflux from J774 macrophages. **Panels A and B.** J774 macrophages were labeled with [^3^H]cholesterol, and treated in the absence (open bars) or presence (closed bars) of cAMP. Lipid-free chimeras or parent apolipoproteins (10 μg/ml) were added to cells in serum-free RPMI-1640 medium, and amount of cholesterol efflux determined at 4 h. Conditions of time and dose were within a linear response range to allow for potential differences (if any) to be observed with and without treatment of acceptors in the absence (**Panel A**) or presence (**Panel B**) of 5x molar excess of BME. **Panel C.** Dependence of cholesterol efflux on chimera concentration. The cells were treated with the indicated concentrations of the chimeras for 4 h. **Panel D.** Kinetics of chimera-mediated cholesterol efflux. Chimera-mediated cholesterol efflux was followed at the indicated time points using 32 μg/μl of each protein. Values are means ±SD from triplicate determinations within a single experiment representative of two. For **Panel C** and **Panel D**, apoE3-NT/apoAI-CT (open inverted triangles); apoAI-NT/apoE-CT (open circles).

Lastly, the relative ability of the lipid-free chimeras to promote ABCA1-mediated cholesterol efflux was examined. ABCA1 is a member of the ATP-binding cassette (ABC) family of transmembrane transporters, which mediates active transport of cholesterol. It bears phospholipid translocase activity that leads to simultaneous efflux of phospholipid and cholesterol to lipid-free or lipid-poor apoAI [42] leading to HDL biogenesis. In the current assay, cholesterol loaded J774 cells were treated with or without cpt-cAMP to modulate expression of ABCA1, followed by treatment with the lipid-free apolipoproteins. In general, both chimeras retained the ability to mediate cholesterol efflux in an ABCA1 dependent manner, Fig 7**, Panel A;** the presence of β-mercaptoethanol (BME) did not have a major influence on the efflux ability, Fig 7**, Panel B**. Interestingly, apoAI-NT/apoE-CT appeared to bear a greater capacity to mediate efflux, compared to either apoE3-NT/apoAI-CT or both parent proteins. With both chimeras, the efflux ability was dose dependent, Fig 7**, Panel C**, and increased as a function of time, Fig 7**, Panel D**. The K_m_ values for apoE3-NT/apoAI-CT and apoAI-NT/apoE-CT are 20 and 32 μg/mL, respectively, and the corresponding V_max_ values are 17 and 19% / 4h. The K_m_ values of the chimeras are 6–7 fold higher compared to the parent proteins, while the V_max_ is twice as high compared to apoAI or apoE3 reported previously [19, 43]. This is indicative of a lower specificity for the chimeras, but suggest the potential to operate at higher concentrations at which apoAI and apoE would have reached saturation.

## Discussion

The strategy of generating chimeras has been adopted widely in protein engineering to examine structure-function relationships and to define regions within a domain or at the subdomain level in closely related proteins. In the case of the family of exchangeable apolipoproteins, the presence of amphipathic *α*-helices is a defining feature among the members; from a structural perspective the amphipathic *α*-helices offer the ability for these proteins to exist in a lipid-free or lipid associated state by undergoing a reversible conformational change, for which lipids are believed to be triggers. From a functional perspective, they serve a variety of functions such as interaction with lipids, lipoproteins and lipoprotein receptors, facilitation of cholesterol efflux promoted by membrane transporters and as enzyme activators. The focus of this study was on apoE3 and apoAI, two key members of the exchangeable apolipoprotein family; the former bears two defined NT and CT domains; the latter also has an NT and CT domain, though the CT domain may remain relatively unstructured at low concentrations (lower than that used in this study), thereby deviating from the conventional definition of a domain. Despite their broad structural similarity, there are significant differences in functionality between the two proteins: whereas apoE3 can bind and mediate cellular uptake of lipoproteins via the LDLr, apoAI lacks this ability. Conversely, whereas apoAI mediates phospholipid vesicle solubilization to yield discoidal particles very rapidly, apoE3 is relatively slow in its ability to cause this transformation, though it would do so given time and under specified experimental conditions. Both proteins show significant ability to promote ABCA1-mediated cholesterol efflux. Our objective was to understand the interaction between and influence of the domains on each other in the two proteins. To achieve this, the CT domain or tail of each protein was swapped to generate apoE3-NT/apoAI-CT and apoAI-NT/apoE-CT.

### Design of chimeric apoE3-NT/apoAI-CT and apoAI-NT/apoE-CT

The splice site for apoE3 was determined based on consensus from a series of biochemical and biophysical studies involving susceptibility to protease cleavage, protein unfolding studies using chemical and heat denaturation, kinetic and equilibrium studies of association-dissociation behavior of apoE [44] and, C-terminal truncation analysis [14, 45, 46]; in addition, it was guided by the high resolution X-ray structure of residues 1–191 [13], NMR structure of 1–183 [47] and, to a limited extent from the NMR analysis of an engineered form of apoE3 (F257A/W264R/V269A/L279Q/V287E) [23] that was generated to obtain a monomeric protein for structural analysis. Taken together, apoE3 NT domain was defined to encompass residues 1–191, and the CT domain to encompass residues 201–299 and the protease sensitive loop residues 192–200.

For apoAI, the splice site was determined broadly based on the boundaries defined by HDX studies on lipid free apoAI carried out by other researchers [48] due to lack of availability of a high resolution structure of the full length protein. X-ray analysis of apoAI truncated at the CT end to yield 1–185 [49] or at the NT end to yield 44–243 [50] provided limited guidance, both suggesting an organization resembling the lipid-associated conformation of the protein in a discoidal complex with phospholipids. Further, truncation at both ends appears to yield a stable helix bundle conformation [51]. The rationale for using HDX data is that it was obtained by non-invasive means and the studies were performed at relatively low concentrations (50–60 μg /ml) wherein the protein was monomeric, comparable and relevant to that used in the current study. At concentrations > 1mg/ml, apoAI self-associates and acquires increased secondary structure [52, 53], presumably due to adoption of helical structure by the CT domain. Taken together, the NT domain was broadly defined to encompass residues 1–180, and the CT domain residues 181–243.

### Biophysical analysis of apoE3-NT/apoAI-CT and apoAI-NT/apoE-CT

Secondary structural analysis revealed that the chimeras are predominantly α-helical, similar to the parent proteins. In both cases, the NT domain appears to play a dominant role in driving the overall fold of the protein. The NT domain of apoE3 bears a helix bundle that is unusually stable among apolipoproteins, showing resistance to GdnHCl- induced unfolding, with a ΔG of unfolding of ~10 kcal/mol [14]. Typically, apoAI and other exchangeable apolipoproteins, including insect apolipophorins, unfold with a [GdnHCl]_1/2_ of ~ 1 M or less, and a ΔG of unfolding of 1–4 kcal/mol. The single transition elicited by apoAI makes it difficult to dissect out the differential folding of its NT and CT domains. By appending the CT tail of apoAI to apoE3 NT domain, which has a much higher stability with a mid point of ~ 2.5 M, the unfolding of apoAI CT tail could be dissected, revealing a less structured state with a low mid point of denaturation (~0.1 M GdnHCl). At the concentration used for the unfolding studies (~0.2 mg/ml) it is possible that a small fraction of apoAI exists as a dimer, in which case, the low values could also represent dissociation of dimeric CT tail. ANS fluorescence analysis further confirmed the presence of less structured CT tail; appending the CT domain of apoE3 to apoAI NT domain, resulted in a much larger increase in ANS fluorescence emission (~100%) compared to that seen when appending the CT tail of apoAI to apoE3 NT domain (~18%). This suggests that the CT domain of apoE3 is far more structured than that of apoAI, a conclusion also supported by the GdnHCl-induced unfolding that revealed a small shift towards a more stable overall structure. The structural flexibility of apoAI CT domain bears direct relevance to its tendency to seek stability with higher lipid-binding propensity compared to apoE3 as discussed later.

Currently available information about apoAI suggests that the CT tail of apoAI bears the propensity to adopt an α-helical structure based on algorithms for secondary structural predictions. This predisposition leads to reference of apoAI CT tail as an additional domain despite its unfolding behavior, which shows a single transition, a characteristic feature of a one-domain protein. The trigger for the transition from flexible to α-helical structure may include: (i) increased protein concentration, which has been shown to lead to apoAI oligomerization, increase in α-helical content and increased cross-linking [48, 52, 53]; (ii) the presence of lipids, which induces helix formation, consistent with the inverse correlation between protein stability and lipid binding ability: unstructured segments seek stability by interacting rapidly with lipids [54]; (iii) truncation of apoAI at the N-terminal end by removing residues 1–43; this resulted in the rest of the protein, including the CT tail, adopting an extended helical structure as noted by X-ray analysis [50]. It is not known if this is due to the high protein concentrations required for X-ray analysis or removal of residues 1–43 that caused the trigger; nonetheless, it points to the facile ability of the CT tail to adopt an α-helical structure.

### Insights obtained from functional analysis of apoE3-NT/apoAI-CT and apoAI-NT/apoE-CT

We, and others, have shown that the isolated CT domain of apoE encompassing residues 201–299, displays significant lipid binding ability [38], whereas the lipid binding behavior of isolated apoE3-NT domain (residues 1–191) resembles that of intact apoE3, showing a poor ability to cause DMPC vesicle solubilization [15]. It is possible that an interaction exists between apoE3 NT and CT domains, wherein the CT domain likely wraps around the NT domain in intact apoE3; such an interaction would either prevent the NT domain helix bundle from opening and interacting with lipids, and/or mask the sites involved in initiating lipid interaction. This conclusion also derives support from the NMR structure of monomeric apoE3, which reveals 3 helices: C1 (W210-S223); C2 (V236-E266); and, C3 (D271-W276) [55, 56]. Nevertheless, if this inference is true, then deleting the CT domain should release the ‘inhibitory’ attenuating effect of the CT domain on the NT domain. The observation that the isolated NT domain displays a poor ability to solubilize DMPC vesicles under these conditions [15] suggests that there are other mechanisms involved in triggering the opening of the NT domain helix bundle.

The apoE3-NT/apoAI-CT chimera offered further insight into the above issue; prior studies from other labs have suggested that residues 44–65 and 220–241 of apoAI bear high lipid binding affinity [3, 57]. Indeed, appending the CT tail of apoAI conferred tremendous lipid binding ability to apoE3 as inferred from the efficiency with which apoE3-NT/apoAI-CT solubilized lipid vesicles, attaining rate constants resembling that of intact apoAI. This indicates that apoAI CT domain plays a role not only in initiating lipid interaction but also in triggering the opening of an otherwise stable apoE3 NT helix bundle. Interestingly, the apoAI-NT/apoE-CT chimera displays robust vesicle solubilization ability comparable to that of intact apoAI. This may be attributed to the contributory role of helix 1 in apoAI, supporting early studies that indicate that the high hydrophobicity is linked to its high lipid binding affinity [57, 58]. Also, the presence of several aromatic and hydrophobic residues towards the N-terminal end of apoAI may contribute towards initiating lipid-binding interaction. Alternately, the CT domain of apoE may offer more surface area in terms of binding of more phospholipid molecules, which would aid in solvation of more free cholesterol, a phenomenon suggested to explain the larger discoidal particles formed by apoE3 [42, 59].

Domain swapping also revealed interesting new information regarding the relative cholesterol efflux abilities of apoAI and apoE3; both have the ability to carry out this function [42], albeit to different extents: apoAI shows significantly higher ability to promote efflux compared to apoE3, with several studies pointing to the critical role of the CT tail, especially helix 10, of apoAI in promoting ABCA1-mediated cholesterol efflux [60, 61]. Appending the apoAI CT tail to apoE3 NT domain however did not result in a significant increase in efflux capability compared to apoE3. Conversely, appending apoE CT tail to apoAI conferred superior cholesterol efflux ability to apoAI; this is indicative of a significant role of apoE3 CT domain in cholesterol efflux reaction, corroborating previous studies that demonstrate that the entire CT domain of apoE was able to efflux with the same efficiency as intact apoAI and apoE3 [43, 62]. Indeed, this was the rationale for the development and design of an apoE3 mimetic peptide that used residues 238–266 of apoE3 as a template with modifications to increase the hydrophobicity and acidic nature of the peptide [19, 22, 62]. These studies underscore the importance of apoE CT domain in promoting cholesterol efflux. Our current study further extends its role by demonstrating the possibility of a synergistic effect between apoAI NT domain and apoE-CT domain, since this chimera’s ability was better than either of the parent proteins. Other studies used chimeric constructs of mouse and human apoAI with their NT domains swapped to obtain insight into mechanism of cholesterol efflux ability [63]. They report that swapping the NT domain of human apoAI with that of the NT domain of mouse apoAI increased the efficiency of ABCA1-mediated cholesterol efflux ability of the former and increased the uptake rate of cholesterol by the liver. They attributed the gain of function to the lower stability of mouse NT domain. An independent study reported that a loss noted in the efflux capacity of apoAI C-terminal deletion mutant (Δ190–243) was recovered when a pair of helices from apoAII (1–77) was substituted to generate an apoAI/AII chimera [64]. Together, these studies lend support to the suggestion that the tertiary structure and the organization of helices, and not the amino acid sequence *per se*, is influential in cholesterol efflux.

It is well established that apoE-containing lipoproteins interact with both the LDLr and related family members, and, HDL receptor SR-BI [65], in addition to cell surface proteoglycans such as heparan sulfate proteoglycans. This was reflected in the significantly high level of intracellular immuno- and DiI fluorescence noted for lipid-associated apoE3 and apoE3-NT/apoAI-CT chimera. Since only a very small decrease was noted in DiI fluorescence in the presence of SR-BI/SR-BII antibody, we believe that the LDLr family mediated pathway is a major route for cellular uptake of lipid-associated apoE3 and apoE3-NT/apoAI-CT, with the SR-BI/SR-BII mediated pathway playing a small role in uptake of DMPC/DiI associated apoE3 and apoE3-NT/apoAI-CT. The presence of apoAI-CT tail does not significantly alter intracellular DiI related fluorescence. Using LDLr-deficient CHO cells that were stably transfected with SR-BI, previous reports have shown that the N-terminal region of apoE3 encompassing residues 1–165 bears sufficient determinants for binding SR-BI receptor in the lipid-associated state [66]. Our current studies indicate that the LDLr mediated pathway is the preferred uptake route for apoE3 containing lipoprotein particles. In the case of DMPC/DiI/apoAI and DMPC/DiI/apoAI-NT/apoE-CT, the intracellular DiI fluorescence was visible albeit to a significantly lesser extent compared to apoE3, which was abolished in the presence of SR-B1/SR-BII antibody. This suggests cellular uptake of DMPC/DiI /apoAI and DMPC/DiI/apoAI-NT/apoE-CT via SR-BI/ SR-BII to a small extent. These observations are consistent with previous reports that suggest that the C-terminal tail (186–243) of apoAI does not appear to significantly affect the binding interaction with SR-BI in the POPC-associated state [65, 67]. Taken together, the chimeric approach indicates that the amphipathic helical motif in apoAI or apoE3 is sufficient for interaction with SR-BI/SR-BII interaction, consistent with previous reports that employed truncated variants [65].

The domain swapping data allows us to reflect on the structural and functional basis of the physiological role of apoE3 and apoAI, two key exchangeable apolipoproteins that are both considered as anti-atherogenic proteins. With its structural flexibility and inherently disordered nature of the CT domain, apoAI is well adapted to bind cholesterol, form small lipoprotein complexes that can be rapidly removed from peripheral tissues. The limited ability of apoE3 to bind small vesicles and cause vesicle solubilization under similar conditions is reflective of its inherent preference for binding large lipoprotein particles, with the high stability of the NT domain helix bundle bearing a regulatory role in clearance of the large atherogenic lipoproteins. The helix bundle opening, which confers LDLr binding competency to the particle, is likely triggered by changes in the particle lipid content, signaling its readiness for clearance. More studies are needed to determine the clearance rate of lipoproteins in the plasma following injection of the chimeric apolipoproteins and investigating the reverse cholesterol transport pathways mediated by the chimeras.

## Supporting information

S1 File Fig A: Schematic representation illustrating generation of apoE3-NT/apoAI-CT chimera; Table I. Primer sequences for the construction of apoE3-NT/apoAI-CT chimera; Fig B: Far UV CD spectra of chimeras; Fig C: Non-denaturing PAGE analysis of DMPC/chimeras.
(DOCX)

## Abbreviations

ΔG_D_: free energy change of unfolding
[GdnHCl]_1/2_: midpoint of guanidine hydrochloride denaturation
apoAI: apolipoprotein AI
apoE3: apolipoprotein E3
CT: C-terminal
DMPC: 1,2-Dimyristoyl-*sn*-glycero-3-phosphocholine
DTT: dithiothreitol
HDL: high density lipoprotein
LUV: large unilamellar vesicles
NT: N-terminal
PAGE: polyacrylamide gel electrophoresis
PBS: phosphate buffered saline
SDS: sodium dodecyl sulfate
SR-BI/SR-BII: Scavenger receptor class B type I/type II
TCEP: tris(2-carboxyethyl)phosphine
