# Supplementary material for: Swapping the N- and C-terminal domains of human apolipoprotein E3 and AI reveals insights into their structure/activity relationship
Source: PLoS One. 2017 Jun 23;12(6):e0178346. doi: 10.1371/journal.pone.0178346 (PMC5482431; doi:10.1371/journal.pone.0178346)
Supplement: S1 File — Fig A: Schematic representation illustrating generation of apoE3-NT/apoAI-CT chimera; Table I. Primer sequences for the construction of apoE3-NT/apoAI-CT chimera; Fig B: Far UV CD spectra of chimeras; Fig C: Non-denaturing PAGE analysis of DMPC/chimeras. (DOCX) [file pone.0178346.s001.docx]

*Supporting information*

Swapping the N- and C-terminal Domains of Apolipoprotein E3 and AI Reveals New Insights into their Structure/Activity Relationship

Mark T. Lek,^a†^ Siobanth Cruz,^a†^ Nnejiuwa U. Ibe,^a^ Wendy H. J. Beck, ^a^ John K. Bielicki, ^b^ Paul M. M. Weers^a^ & Vasanthy Narayanaswami*^,a^

^†^Equal author contribution

^a^Department of Chemistry and Biochemistry, California State University Long Beach, CA; ^b^Lawrence Berkeley National Laboratory, Donner Laboratory, MS1-267, Berkeley, CA

**Design and Generation of Chimeric DNA Constructs**. The codon-optimized sequence for apoAI-NT/apoE-CT in pET-20b(+) vector was synthesized commercially (Eurofins MWG Operon, Inc., Huntsville, AL). That for apoE3-NT/apoAI-CT was generated from the codon-optimized sequence of parent apoE3 and apoAI by the overlap extension polymerase chain reaction (PCR) method using overhang primers ([1](#_ENREF_1), [2](#_ENREF_2)) as schematically illustrated in **S1 Fig A**.


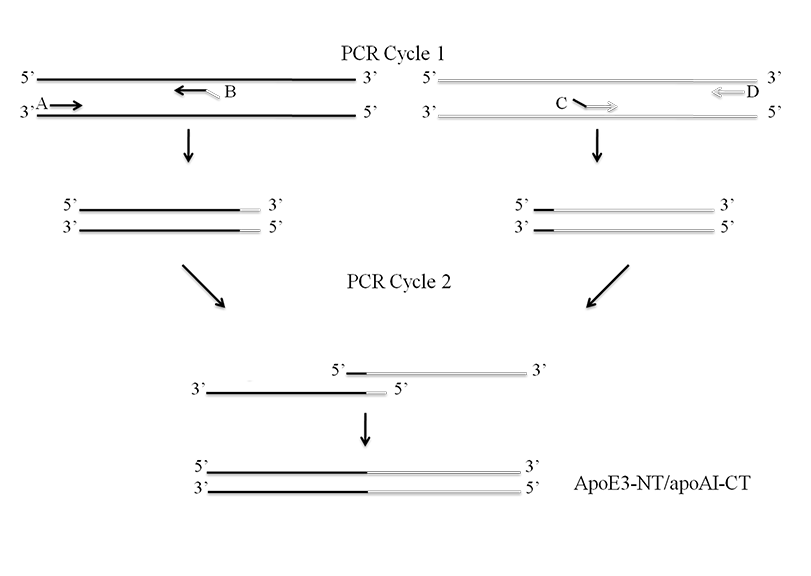


**Fig A:** **Schematic representation illustrating generation of apoE3-NT/apoAI-CT chimera**. The NT (residues 1-191) and CT (residues185-243) segments of apoE3 (black) and apoAI (light grey), respectively, were amplified using overhang primers in the first round of PCR. Primers A and D are T7 promoter and terminator primers, respectively, while B and C represent chimeric primer #1 and #2 (Table ). The amplified segments served as megaprimers in the initial cycles of a second round of PCR with an annealing temperature of 48 °C to populate the spliced chimeric sequence; this was followed by amplification of the entire chimeric coding sequence using T7 primers with an annealing temperature of 53 °C.

In step 1, the coding sequence for: (i) apoE3 NT domain bearing a small segment of the N-terminal portion of apoAI CT domain was amplified by PCR reaction utilizing T7 promoter primer and chimeric primer #1 (**Table I**) as forward and reverse primers, respectively; chimeric primer #1 was designed such that it bears a 5' overhang complementary to the initial segment coding for apoAI CT domain; and, (ii) apoAI CT domain bearing a small segment of the C-terminal portion of apoE3 NT domain was amplified by PCR reaction utilizing chimeric primer #2 (**Table I**) and T7 terminator primer as forward and reverse primers, respectively; chimeric primer #2 was designed such that it bears a 3' overhang complementary to the terminal segment coding for apoE3 NT domain. Each primer was designed so that the final amplified product bears a partial sequence complementary to the desired inserted domain. Both reactions were carried out under standard conditions using *Pfu* Ultra DNA polymerase (Agilent Technologies, Santa Clara, CA). The chimeric primers were synthesized by Eurofin MWG Operon, Louisville, KY. The final amplified products were treated with *Dpn I* to digest the parent bacterial-generated DNA templates and purified using QIAquick PCR Purification Kit (Qiagen, Valencia, CA).

Table I. Primer Sequences for the Construction of apoE3-NT/apoAI-CT chimera

| Chimera Primer | Primer Sequence (5’ 🡪 3’) |
| --- | --- |
| Primer #1: ApoE3-NT | *CCCGCCGTTCTCCTTGAG*ACGGACGCGACCCTGTTCCACGAGCGG |
| Primer #2: ApoAI-CT | *CGCTTGGCCGCGCGCCTTGAGGCT*GCAGCAACAGTTGGGAGCTTGGCG |

*Italics denote overhang nucleotides*

In step 2, the two purified amplified products from step 1 with overlapping complementary sequence from each other were subjected to 2 stages of PCR reaction in the presence of T7 promoter and terminator primers (all other conditions being the same): the first stage involved 4 cycles of amplification with an annealing temperature of 48 °C, while the second stage involved 29 cycles with an annealing temperature of 53 °C. This step concluded with an additional extension time of 5 min at 72 °C. The final amplified products were purified as above.

**Ligation into pET20 b(+) expression vector.** The purified amplified product from step 2 above and the pET-20b(+) vector were subjected to restriction digestion with Hind III and Nde I (New England Biolabs, Inc., Ipswich, MA) at 37 °C for 1 h. The digested products were electrophoresed on a 1% agarose gel, excised and the DNA recovered using Zymoclean Gel DNA Recovery Kit (Zymo Research, Irvine, CA). The recovered DNA were ligated using T4 DNA ligase at 16 °C for 16 h. A portion of the ligated product was used to transform DH5α *E. coli* cells by the heat shock method, and the plasmid sequence was verified (Genewiz, Inc., La Jolla, CA) to ensure correct splicing between the desired segments of apoE3 and apoAI coding sequence.

The chimeric construct bears a protease cleavage site immediately before the first residue of apoE3, preceded by a hexa His-tag to enable purification by affinity chromatography. The protease cleavage site was engineered to facilitate cleavage of the hexa His-tag following purification. However, for the purposes of the current study, the hexa-His tag and the protease cleavage site remained in the expressed protein, as they did not affect the overall fold or function of the proteins. A similar strategy was attempted for generating the apoAI-NT/apoE-CT chimera, but was without success. The codon optimized sequence for this construct (apoAI residues 1-180 followed by apoE (192-299) was synthesized commercially along similar lines, and ligated into a pET-20b(+) vector. In both cases the nucleotide sequence was verified to confirm the presence of the desired segments of the parent proteins in the spliced DNA. Upon transformation in *E. coli* BL21-Gold (DE3) pLys, and induction with IPTG, both constructs yielded significant levels of protein expression, with yields of purified protein ranging between 20 and 40 mg per liter culture medium.

**Circular dichroism spectroscopy.** The ellipticity of the samples was recorded between 185 and 260 nm on a Jasco 810 spectropolarimeter (Jasco Inc., Easton, MD) using a 1.0 mm path length cylindrical cuvette (Hellma Cells, Plainview, NY). The proteins were dissolved at a concentration of 0.2 mg/ml in 10 mM ammonium bicarbonate buffer, pH 7.4. Four independent scans were averaged after recording at a scan speed of 50 nm/min, with a response time of 1 s and bandwidth of 1 nm at 24 °C. The molar ellipticity [*θ*] in deg cm^2^ dmol^-1^ at 222 nm was obtained using the equation:

[*θ*]_222nm_  = MRW(*θ*) / (10*lc*)

where MRW is the mean residue weight, *θ* is the measured ellipiticity at 222 nm (deg), *l* is the cuvette path length (cm), and *c* is the protein concentration (g/mL). The percent *α*-helical content was calculated as described previously ([3](#_ENREF_3)) from the molar ellipticity values using:

% *α*-helix = {( - [*θ*]_222_ + 3000) / 39000 x 100


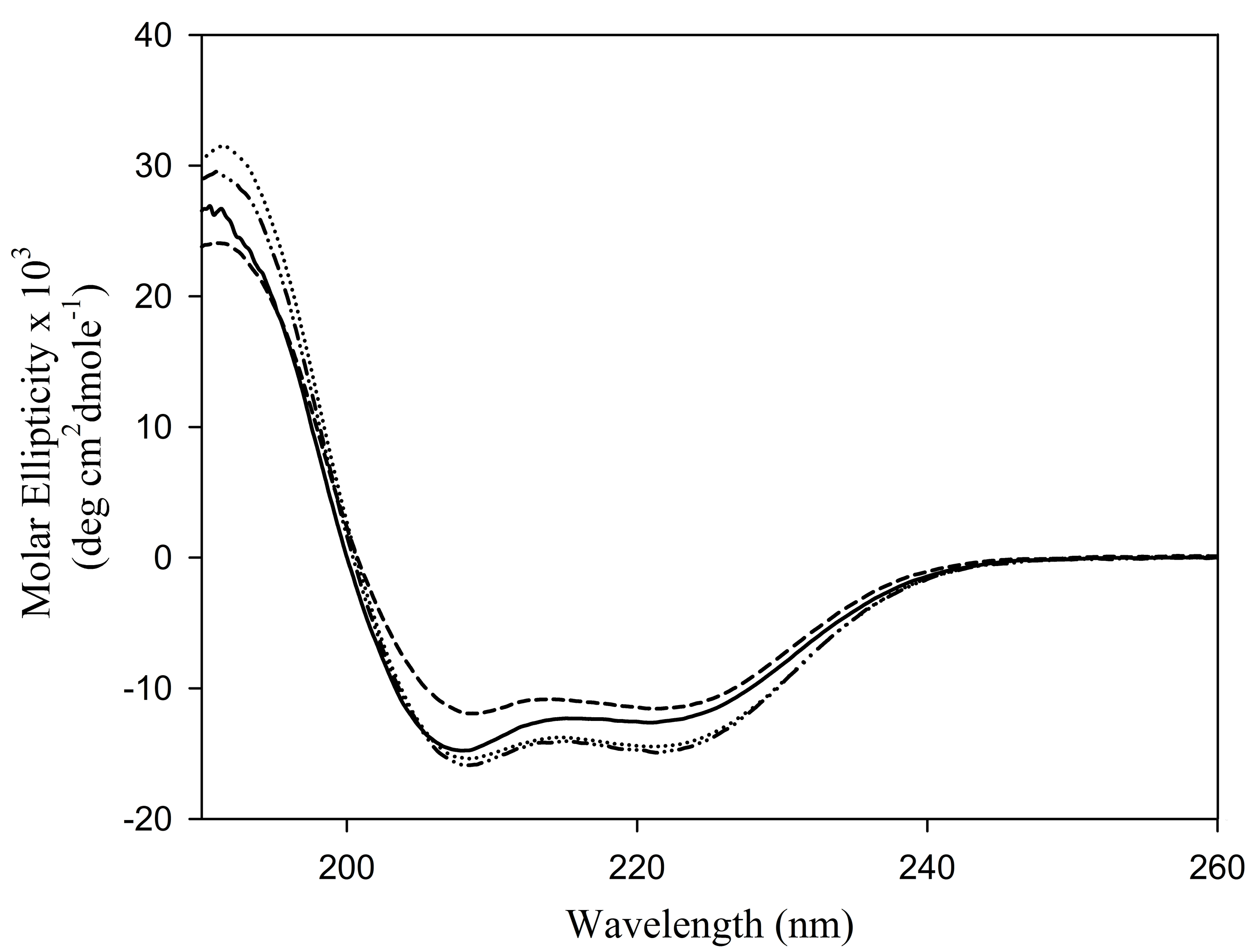


**Fig B: Far UV CD spectra** **of chimeras**. The spectra of the chimeric and parent apolipoproteins (0.2 mg/ml in 10 mM ammonium bicarbonate buffer, pH 7.4) were recorded between 185 and 260 nm in a 1.0 mm path length cuvette. Four scans were averaged after recording at a scan speed of 50 nm/min at 24 °C. ApoAI ( _____ ); apoAI-NT/apoE-CT (·······); apoE (-----); and apoE3-NT/apoAI-CT (··-··-··).

**Lipid Binding Assay**. MLVs were prepared by initially forming a thin film of DMPC (10 mg DMPC dissolved in 1 ml of 3:1 (v/v) mixture chloroform: methanol) under a stream of N_2_, incubating at 42 ^o^C 1 ml PBS, and vigorously vortexing for 30 s with three intervals. Vesicle solubilization was initiated by addition of 125 µg of apolipoprotein or buffer to 125 µg of DMPC MLVs that have been previously equilibrated in 400 µl of PBS in a cuvette held at 23.9 ^o^C in a Peltier-controlled PerkinElmer LS 50B spectrometer. The contents were mixed thoroughly and the change in absorbance at 325 nm was measured for 30 min. The data plots were normalized to the initial absorbance value at 325 nm.

**Preparation of DMPC-bound complexes of chimera or parent proteins**. The DMPC-bound complexes were prepared by the sonication method as described previously (4). The particle size was estimated by non-denaturing PAGE analysis using 4–20% acrylamide gradient Tris-glycine gels (loading~ 10 μg protein). Electrophoresis was carried out in the presence of protein standard markers (Amersham HMWCalibration Kit, G.E. Healthcare) for 18 h at 132 V at 4°C, and the gels stained with Instant Blue (Expedeon Inc., San Diego, CA). Particle composition was determined by carrying out protein (Dc kit, BioRad Laboratories, Hercules, CA) and phospholipid (Wako Chemicals USA, Inc., Richmond, VA) assays.


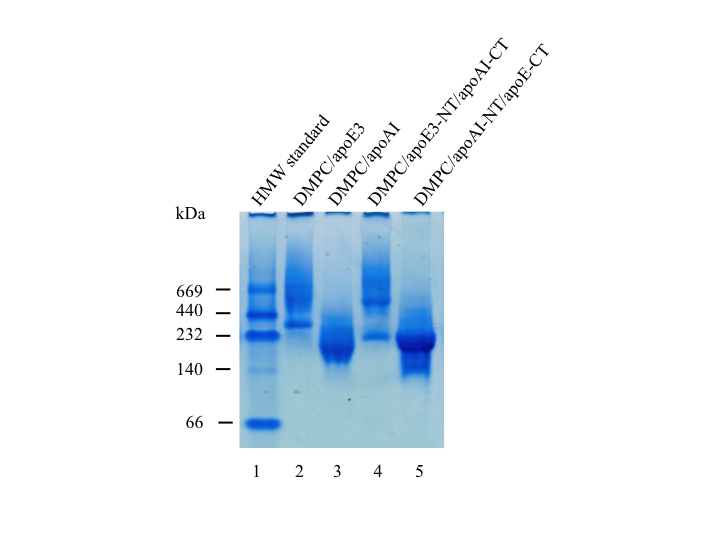


**Fig C. Non-denaturing PAGE analysis of DMPC/chimeras and DMPC/parent proteins.** Non-denaturing PAGE of DMPC-bound complexes of chimeras and parent proteins (10 μg protein) was carried out using 4-20% acrylamide gradient Tris-glycine gels. Electrophoresis was carried out in the presence of protein standard markers (Lane 1) (thyroglobulin (669 kDa), ferritin (440 kDa), catalase (232 kDa), lactate dehydrogenase (140 kDa) and bovine serum albumin (66 kDa) bearing Stokes’ diameter of 17, and 12.2, 10.4, 9.3 and 7.1 nm, respectively) for 18 h at 132 V at 4°C, and the gels stained with Instant Blue. The lane assignments are: Lane 2: DMPC/apoE3; Lane 3: DMPC/apoAI; Lane 4: DMPC/apoE3-NT/apoAI-CT; Lane 5: DMPC/apoAI-NT/apoE-CT.

**Cellular uptake of lipoproteins by glioblastoma cells.** Glioblastoma cells were cultured in DMEM with 10 % fetal bovine serum (FBS), 5000 IU/mL penicillin and 5000 µg/mL streptomycin sulfate at 37 °C according to ATCC guidelines. For uptake experiments, the cells were grown to ~60 % confluency on a cover glass, placed in a 6-well cell culture cluster (~ 1x10^6^ cells per well), washed with pre-warmed medium containing 10 % LPDS and incubated for 24 h to induce LDLr expression. The cells were then treated with 0 or 10 µg of DMPC-bound complexes of the chimeras or parent proteins in 10% LPDS medium and incubated for 2 h at 37 ^o^C. The DMPC-bound complexes were prepared as described above.

Cellular uptake of the lipoprotein particles was followed by immunofluorescence using monoclonal antibodies targeted against the NT or CT domain of apoE, 1D7 or 3H1, respectively, (in PBS, 1:3000 dilution) and Alexa555-labeled secondary antibody.

Uptake of the lipid components was visualized by direct fluorescence using DMPC/parent or chimeric protein complexes containing 1% 1,1'-dioctadecyl-3,3,3',3'-tetramethylindocarbocyanine iodide (DiI, Invitrogen Life Technologies, Grand Island, NY). DiI was incorporated into DMPC complexes by addition from a stock solution of DMSO and incubation at 37°C for 18 h in dark. Unbound DiI was separated from lipoprotein-bound DiI by density gradient ultracentrifugation. The top fractions containing DMPC/DiI/protein were pooled and dialyzed against PBS. Cellular uptake was followed as described above following addition of 0.5 µg/ml of DMPC/DiI/complexes of the chimeras or parent proteins. The cells were stained with DAPI (4',6-Diamidino-2-phenylindole dihydrochloride) in PBS to visualize the nucleus. To determine the involvement of SR-BI, the receptor was blocked by treating the cells with SR-BI/SR-BII antibody (NB400-104, Novus Biologicals) (1:500 dilution) in the presence of DMPC/DiI/complexes of the chimera or parent proteins as described above.

The cells were visualized by confocal laser scanning microscopy (Olympus IX-81) with 405 laser for DAPI, 559 for Alexa 555 and 559 for DiI, and the images captured via Olympus Fluoview 1000.

**Cholesterol efflux assay**. The ability of apolipoproteins to mediate cellular cholesterol efflux was assessed using J774 mouse macrophages. Cells were treated with and without 8-(4-chlorophenylthio)adenosine 3':5'-cyclic monophosphate (cpt-cAMP), an analog of cAMP to modulate ABCA1 expression. Apolipoprotein acceptors were used in lipid-free form. Briefly, J774 cells were plated onto 24-well culture plates in RPMI-1640 culture medium containing 10% FBS. Two-days before cholesterol efflux was assessed, the cells were labeled with 1 Ci [^3^H]cholesterol/ml of RPMI-1640 culture medium containing 1% FBS; cpt-cAMP was added the day prior to experiments (20 h treatment) to up-regulate ABCA1 expression. Cells were next rinsed with serum-free 1640 RPMI culture medium, incubated for 30 min with medium containing 0.2% BSA, and subsequently washed twice with serum-free medium. Apolipoproteins prepared fresh in PBS were diluted in serum-free RPMI-1640 medium and added directly to rinsed cells and incubated for 4 h. The amount of [^3^H]cholesterol appearing in the medium was quantified by liquid scintillation counting. The [^3^H]cholesterol appearing in the medium was normalized to cellular content of [^3^H]cholesterol prior to initiation of efflux, a value obtained from parallel sets of wells. In some experiments, stock solutions (1 mg/ml) of apolipoproteins were treated with a 5-fold molar excess of BME relative to apolipoprotein stock concentration for 16 h at 4^o^ C, then diluted in serum-free RPMI-1640 medium immediately before addition to cells for assessment of lipid efflux activity.

**References**

1. Perez K, Yeam I, Jahn MM, & Kang BC (2006) Megaprimer-mediated domain swapping for construction of chimeric viruses. *J. Virol. Meth.* 135(2):254-262.

2. Wei D, Li M, Zhang X, & Xing L (2004) An improvement of the site-directed mutagenesis method by combination of megaprimer, one-side PCR and DpnI treatment. *Anal. Biochem.* 331(2):401-403.

3. Morrow JA*, et al.* (2000) Differences in stability among the human apolipoprotein E isoforms determined by the amino-terminal domain. *Biochemistry* 39(38):11657-11666.

4. Kim SH, Adhikari BB, Cruz S, Schramm MP, Vinson JA, Narayanaswami V (2015) Targeted Intracellular Delivery of Resveratrol to Glioblastoma Cells Using Apolipoprotein E-Containing Reconstituted HDL as a Nanovehicle. *PLOS One* 10, e0135130
